# Isosorbide and 2,5-Furandicarboxylic Acid Based (Co)Polyesters: Synthesis, Characterization, and Environmental Degradation

**DOI:** 10.3390/polym14183868

**Published:** 2022-09-15

**Authors:** Chaima Bouyahya, Rafael Patrício, Ana Paço, Mafalda S. Lima, Ana C. Fonseca, Teresa Rocha-Santos, Mustapha Majdoub, Armando J. D. Silvestre, Andreia F. Sousa

**Affiliations:** 1 CICECO—Aveiro Institute of Materials, Department of Chemistry, University of Aveiro, 3810-193 Aveiro, Portugal; 2 Laboratoire des Interfaces et Matériaux Avancés, Université de Monastir, Monastir 5000, Tunisia; 3 Centre for Environmental and Marine Studies (CESAM), Department of Chemistry, University of Aveiro, 3810-193 Aveiro, Portugal; 4 Centre for Mechanical Engineering, Materials and Processes, Department of Chemical Engineering, University of Coimbra Rua Sílvio Lima—Polo II, 3030-790 Coimbra, Portugal

**Keywords:** isosorbide, 2,5-furandicarboxylic acid, biobased (co)polyesters, environmental degradation

## Abstract

Poly(2,5-furandicarboxylate)s incorporating aliphatic moieties represent a promising family of polyesters, typically entirely based on renewable resources and with tailored properties, notably degradability. This study aims to go beyond by developing poly(isosorbide 2,5-furandicarboxylate-*co*-dodecanedioate) copolyesters derived from isosorbide (Is), 2,5-furandicarboxylic acid (FDCA), and 1,12-dodecanedioic acid (DDA), and studying their degradation under environmental conditions, often overlooked, namely seawater conditions. These novel polyesters have been characterized in-depth using ATR-FTIR, ^1^H, and ^13^C NMR and XRD spectroscopies and thermal analysis (TGA and DSC). They showed enhanced thermal stability (up to 330 °C), and the glass transition temperature increased with the content of FDCA from ca. 9 to 60 °C. Regarding their (bio)degradation, the enzymatic conditions lead to the highest weight loss compared to simulated seawater conditions, with values matching 27% vs. 3% weight loss after 63 days of incubation, respectively. Copolymerization of biobased FDCA, Is, and DDA represents an optimal approach for shaping the thermal/(bio)degradation behaviors of these novel polyesters.

## 1. Introduction

Public awareness concerning the accumulation of polymers (commonly known as *plastics*) in landfill and aquatic environments is increasing as a result of a lack of effective waste management and systems worsened by limited information about their behavior under environmental conditions [1,2,3,4,5,6]. Additionally, the fact that most of the currently used polymers are synthesized from dwindling and polluting fossil feedstocks [7,8] is also of major general concern. These two issues have driven the need to, on the one hand, focus on the development of (bio)degradable polymers, especially for specific fields of application, and, on the other hand, to shift to the use of biobased polymers as a replacement for those derived from fossil resources. These approaches will ultimately contribute to reducing the current global plastic pollution and lead to more sustainable development [5,7,8,9,10].

Recently, biobased aliphatic-(hetero)aromatic polyesters have received much interest because of their ability to behave as high-performance materials possessing target (bio)degradability, which may be fine-tuned by simply adjusting the relative proportion of the aliphatic and the aromatic fractions [11,12,13]. In this regard, Sousa et al. [11] studied the degradation and thermal properties of a set of copolyesters prepared from 2,5-furandicarboxylic acid (FDCA) by the two-step bulk polytransesterification reaction. FDCA is a prominent bio-derived monomer for polyester synthesis. Poly(ethylene 2,5-furandicarboxylate)-co-poly(lactic acid)s incorporating only 8 mol% of lactyl units have excellent degradability while displaying very high thermal stability and high glass transition (*T_g_*) values (*ca*. 324 and 76 °C, respectively) similar to those of the PEF homolog. Another study reported the poly(1,20-eicosanediyl 2,5-furandicarboxylate), also prepared by bulk polytransesterification, which has economic interest for food packaging applications and promising degradability under hydrolytic conditions [12]. Other authors explored the degradation of copolymers of FDCA with succinic acid [14], dimerized fatty acids [15], or diglycolic acid [16], among several others. However, the properties of these materials can be advantageously improved if biobased 1,4:3,6-dianhydro-D-glucitol, also known as isosorbide (Is), was used as a building-block component. Indeed, the presence of isosorbide, owing to its molecular structure rigidity, good thermal stability, stereochemical properties, and hydrophilicity, can impart improved glass transition temperature and hydrolytic and/or (bio)degradability properties to such polymers [17,18,19,20,21,22]. In this vein, Lomelí-Rodríguez et al. [23] studied the effect of isosorbide on aliphatic polyester coatings, demonstrating that the introduction of 50 mol% of this monomer into the polyester backbone increased the *T_g_* from 10 to 53 °C. The degradability of isosorbide and aliphatic dicarboxylic acid (e.g., succinyl, glutaryl, and adipoyl dichlorides)-based polyesters has also been explored [24,25,26]. Bikiaris and colleagues [17], studied the poly(decamethylene 2,5-furandicarboxylate)-co-(isosorbide 2,5-furandicarboxylate) copolyesters. The syntheses of these copolyesters were based on the polytransestrification of bis(hydroxyisosorbide)-2,5-furandicarboxylate and bis(hydroxydecamethylene)-2,5-furandicarboxylate monomers, enabling thus that all added isosorbide and 1,10-decanediol moieties were incorporated in the copolymer backbone. At low isosorbide content (5–20 mol%), the copolyesters show an improvement in mechanical performance with respect to related poly(decamethylene 2,5-furandicarboxylate) homopolyester, reaching Young’s modulus of 559 MPa (15 mol% of Is). Degradation studies in soil revealed that weight loss tends to increase with increasing decamethylene moiety molar ratio in the copolyester main chain. More recently, the same authors studied a poly(isosorbide furandicarboxylate-co-azelate) series of copolyesters [27]. However, the degradation of these polymers in seawater or other aquatic conditions remains unknown.

In this context, the preparation of novel biobased polyesters from FDCA, Is, and dodecanedioic acid (DDA) by bulk polytransesterification and an assessment of their degradation in an aqueous environment has been accomplished. The choice of these monomers is associated, apart from the renewable origin of all these monomers and the previewed polymers’ enhanced properties, and the fact that they are original, with their commercial availability (or very near to the market). In the particular case of the linear aliphatic unit, DDA is readily accessible by oxidation of *Vernonia galamensis* oil [28,29] and is commercially available. It is used in a wide variety of applications, including powder coatings, adhesives, paint materials, corrosion inhibitors, and surfactants [30,31]. The novel (co)polyesters have been characterized in detail in terms of their structure (FTIR, ^1^H, and ^13^C NMR spectroscopies), thermal properties (DSC, DMTA, and TGA), and the extent of degradation under different hydrolytic conditions assessed gravimetrically and by analyzing the exposed surface of the films using scanning electron microscopy (SEM).

## 2. Materials and Methods

### 2.1. Materials

2,5-Furandicarboxylic acid (FDCA, 98%) was purchased from TCI Chemicals (Zwijndrecht, Belgium). 1,12-Dodecanedioic acid (DDA 98%) was supplied by Fluka (Oeiras, Portugal). Dibutyltin(IV) oxide (DBTO) was purchased from TEGOKAT 248. Isosorbide (Is, 99%), titanium(IV) isopropoxide (Ti(OiPr)_4_, 99.999%), deuterated chloroform (CDCl_3_, 99.5 atom % D), lipase from porcine pancreas (PPL), and sea salts (NutriSelect^TM^ Basic) were obtained from Sigma–Aldrich (Oeiras, Portugal). Methanol (analytical grade), chloroform (HPLC grade), and sodium phosphate monobasic anhydrous (NaH_2_PO_4_) were provided by Fisher Scientific (Porto Salvo, Portugal). Sodium phosphate dibasic (Na_2_HPO_4_, 99+%) was acquired from ACROS Organics (Porto Salvo, Portugal). All chemicals and solvents were used as received without further purification.

### 2.2. Synthesis of Dimethyl 2,5-Furandicarboxylate (DMFDC)

The synthesis of DMFDC followed a previously reported procedure [32]. Briefly, DMFDC was prepared by allowing to react FDCA with an excess of methanol under acidic conditions at 80 °C for approximately 15 h. The reaction mixture was allowed to cool down to room temperature, and the ensuing white precipitate was isolated by filtration, thoroughly washed with cold methanol, and dried.

### 2.3. Synthesis of Poly(Isosorbide 2,5-Furandicarboxylate-Co-dodecanedioate) (Co)Polyesters (PIsFDDs)

PIsFDD (co)polyesters were synthesized by bulk polyesterification reactions using ca. 1.0 g of DMFDC, a variable amount of DDA (DMFDC/DDA molar ratios = 100/0, 90/10, 70/30, 60/40, 20/80, 10/90, and 0/100), and a stoichiometric amount of isosorbide. Polymers prepared from higher molar ratios of DMFDC (DMFDC/DDA molar ratios = 100/0, 90/10, 70/30, and 60/40) were synthesized as previously reported elsewhere [12], starting with a molar ratio of DMFDC + DDA/Is = 1/2.05, and using Ti(OiPr)_4_ as catalyst (0.1 wt% relative to the total monomer weight). In the first step, the reaction mixture was progressively heated for 4 h from 160 °C to 170 °C under a nitrogen atmosphere. Afterward, the reaction medium was kept at 160 °C, and an additional amount of DMFDC + DDA was added (molar ratio of 1/1.05). Subsequently, the temperature was raised to 190 °C for 4.5 h under a nitrogen atmosphere. In the second step, vacuum (~10^−3^ mbar) was applied, and the temperature raised up to 250 °C. Then, the mixture was dissolved in chloroform with some drops of TFA, and the polymers were precipitated by pouring the solution into an excess of cold methanol, filtered, and dried.

Polymers prepared with lower amounts of DMFDC (DMFDC /DDA molar ratios = 0/100, 10/90, 20/80) were synthesized using a similar procedure as just described, except that the maximum temperature allowed to reach in the third step was between 210–220 °C (6 h). Polymer films were prepared by slowly melting their powder up to a maxim temperature of 185 °C in a mold and then, cutting them into square shape specimens (*ca.* 0.5 × 0.5 cm^2^).

### 2.4. Characterization Techniques

Attenuated total reflectance Fourier transform infrared spectroscopy (ATR FTIR) spectra were obtained using a PARAGON 1000 Perkin Elmer FTIR Spectrophotometer (Waltham, MA, USA) equipped with a single horizontal Golden Gate ATR cell. The spectra were recorded at a resolution of 8 cm^−1^ and 128 scans in the spectral region of 500–4000 cm^−1^.

^1^H and ^13^C Nuclear Magnetic Resonance spectroscopy (^1^H, ^13^C NMR) analyses of samples dissolved in CDCl_3_ were recorded using a Bruker AMX 300 spectrometer (Wissembourg, France), operating at 300 and 75 MHz, respectively. All chemical shifts were expressed in parts per million (ppm) using tetramethylsilane (TMS) as the internal reference.

Intrinsic viscosity [*η*] measurements of (co)polyesters were performed using an Ubbelohde type viscometer, at 25 °C, in a mixture of phenol/1,1,2,2-tetrachloroethane (50/50 wt%/wt%). All (co)polyesters were dissolved in that solvent mixture (0.1 g per 20 mL) and kept at 140 °C for 20 min to achieve complete dissolution. The intrinsic viscosity was determined by the ratio of specific viscosity and samples solution concentration: $\eta_{\mathrm{sp}}/C$, where $\eta_{\mathrm{sp}}=\left(t_{1}-t_{0}\right)⁄t_{0}$, and *t*_0_ and *t*_1_ are the solvent mixture elution time of the solvent mixture and (co)polyesters solutions, respectively.

Differential Scanning Calorimetry analysis (DSC) thermograms were obtained with a Pyris Diamond DSC calorimeter from Perkin Elmer (Waltham, MA, USA), using nitrogen as purging gas (20 mL·min^−1^) and aluminum pans to encapsulate the samples (*ca.* 5 mg). The calorimeter was calibrated for heating temperature with approximately 10 mg of each of the following metals: 99.999% pure indium, *T_m_* =156.60 °C, and 99.999% pure lead *T_m_* = 327.47 °C. Scans were conducted with a heating rate of 10 °C·min^−1^ in the temperature range of −40 to 250 °C.

Thermogravimetric analyses (TGA) were carried out with a Shimadzu TGA50 analyzer (Kyoto, Japan) equipped with a platinum cell, using platinum pans to encapsulate the samples. Thermograms were recorded under a nitrogen flow (20 mL·min^−1^) and heated at a constant rate of 10 °C·min^−1^ from room temperature up to 800 °C.

Dynamic Mechanical Thermal Analysis (DMTA) of thick samples (10.0 × 5 × 1 mm^3^), placed in a foldable stainless-steel materials pocket of Triton technology, was performed with a Tritec 2000 DMTA Triton equipment (WA, USA) operating in the single cantilever bending geometry. Tests were carried out in multifrequency mode (1 and 10 Hz), from −80 to 250 °C, at 2 °C·min^−1^. The *T_g_* of the PIsFDD (co)polyesters were determined from the maximum of Tan δ vs. T curve, at 1 Hz.

X-ray diffraction (XRD) analyses were carried out using the Philips X’pert MPD instrument operating with CuKα radiation (λ = 1.5405980 Å) at 40 kV and 50 mA. Samples were scanned in the 2*θ* range of 5 to 70°, with a step size of 0.026° and time per step of 67 s.

Scanning electron microscopy (SEM) images of the surface of the films were acquired using a field emission gun-SEM Hitachi SU70 microscope (Tokyo, Japan) operating at 4 kV. Samples were deposited onto a sample holder and coated with evaporated carbon twice.

### 2.5. PIsFDDs Degradation under Simulated Sea Water, Enzymatic, and PBS Hydrolytic Conditions

In vitro simulated seawater experiments were carried out using square-shape specimens (0.5 × 0.5 cm^2^ of ca. 16–31 mg) of (co)polyesters (PIsDD, of PIsF, PIsFDD 60/40 and 10/90) incubated on 10 mL of NutriSelect^TM^ Basic sea salts’ solution (35 g·L^−1^, pH 8.2) at ca. 18 °C. Pure hydrolytic and enzymatic experiments were carried out with film specimens (0.5 × 0.5 cm^2^ of ca. 16–31 mg), using 10 mL of a phosphate buffer solution (0.1 M, pH 7.4), additionally having porcine pancreas lipase (1 mg·mL^−1^) in the case of enzymatic degradation tests, and incubated at 37 °C for 63 days. The specimens were taken out from the solutions at regular intervals (each 3 or 7 days), rinsed thoroughly with distilled water, dried under vacuum for 3 days, and weighed. In order to prevent saturation, the buffer solution was renewed every 7 days. The weight-loss percentage was calculated as follows: $weight\;loss\;\left(\%\right)=\left[\left(w_{0}-w_{d}\right)/w_{0}\times 100\right]$, where $w_{0}$ and $w_{d}$ stand for the initial and prior incubation specimens’ weight, respectively. Each experiment was carried out in triplicate.

## 3. Results and Discussion

**Scheme 1:**
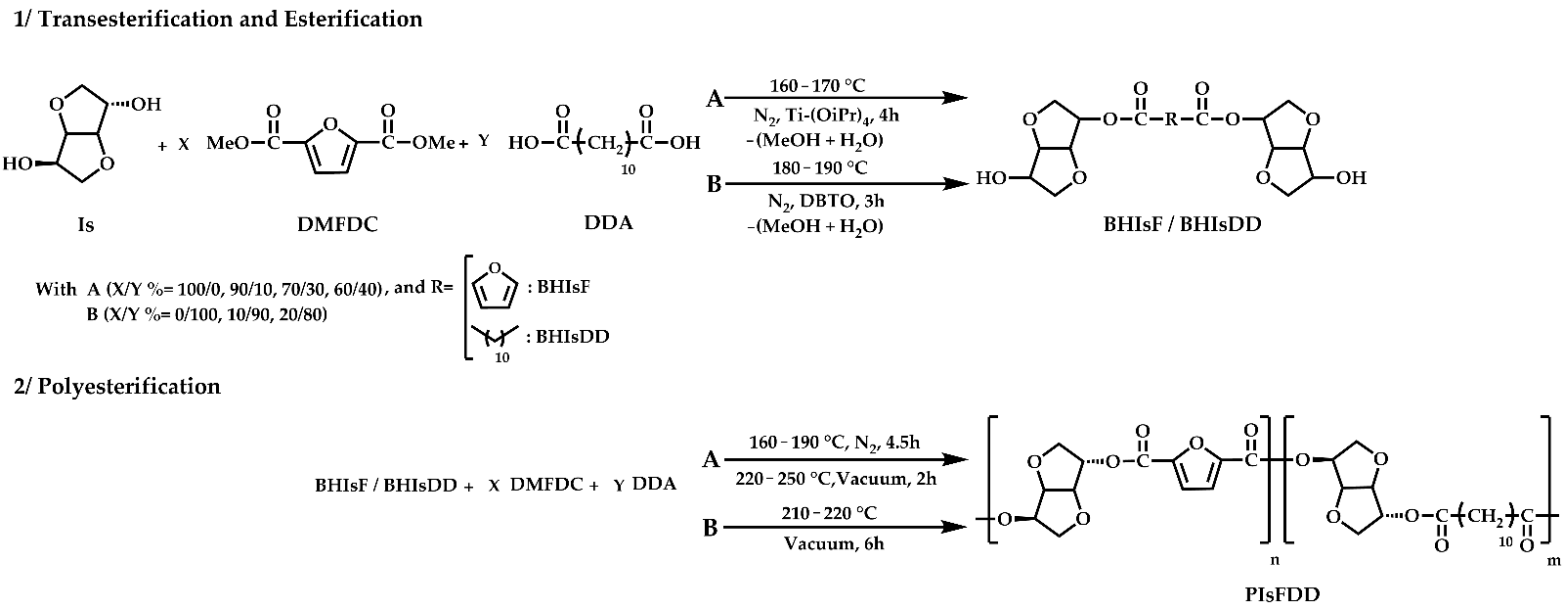
Synthesis of poly(isosorbide 2,5-furandicarboxylate)-*co*-(isosorbide 1,12-dodecanedioate) copolyesters.

A series of fully biobased poly(isosorbide 2,5-furandicarboxylate)-co-(isosorbide 1,12-dodecanedioate) (PIsFDD) (co)polyesters were herein prepared for the first time using biobased monomers, namely isosorbide, dimethyl 2,5-furandicarboxylate (FDCA derivate), and 1,12-dodecanedioic acid and the conditions described in Scheme 1. These new polymers have both stiff and soft units, owned to the furanic and isosorbide moieties and to 1,12-dodecanedioate units, respectively, thus tailoring the properties of the resulting materials, most notably their thermal as well as (bio)degradation behavior. The (co)polyester isolation yields, after purification, were around ~60%, and their intrinsic viscosities were near 0.2 dL·g^−1^. Detailed values for each polymer are summarized in Table 1.

### 3.1. Structural Characterization of PIsFDDs

**Figure 1:**
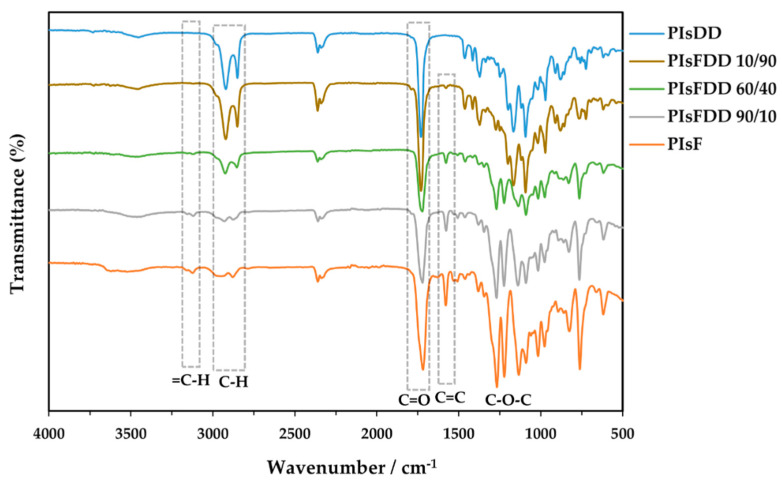
ATR-FTIR spectra of selected PIsFDD copolyesters and related PIsF and PIsDD homopolyesters.

The elucidation of the expected chemical structure of the newly prepared (co)polyesters was confirmed by ATR FTIR spectroscopy. Accordingly, the spectra of Figure 1 display a very intense band near 1723 cm^−1^, assigned to the C=O stretching vibration, characteristic of ester groups, and another typical band at 1271 cm^−1^, assigned to the C-O-C stretching mode also from ester groups. The presence of these two bands, as well as the absence of a noticeable OH bond stretching near 3400 cm^−1^, confirms the success of these polymerizations. It is also observed that the relative intensity of the two bands near 2976 and 2820 cm^−1^ (anti-symmetrical and symmetrical C-H stretching modes) increased with the content of 1,12-dodecanedioate (DD) in the (co)polyester backbone, as evidenced in Figure 1 spectra. Oppositely, the relative intensity of the characteristic absorption bands of the furan ring, at 3130 cm^−1^ (=C-H) and 1575 cm^−1^ (C=C), decreased instead with the DD amount increment (or furan units decrease). The ATR FTIR spectra of all (co)polyesters are available as Electronic Supplementary Material (Figure S1 of supplementary material).

The ^1^H NMR spectra (Figure S2 of supplementary material) confirm the expected basic structure of the furan-based polyesters (PIsF and PIsFDDs), displaying, accordingly, a resonance ascribed to the furan ring protons (*δ* ~ 7.25 ppm). In the case of those polymers incorporating 1,12-dodecanedioate units (PIsDD and PIsFDDs), their spectra show several typical proton resonances at (*δ* ~ 2.33, 1.63, and 1.27 ppm attributed to the various methylene groups (C(O)C*H_2_*, C(O)CH_2_C*H_2_* and mid-chain C*H_2_*, respectively). All spectra show the isosorbide methylene proton resonances, split into four multiplets in the neighborhood of furan moiety, at *δ* ~ 5.40, 5.07, 4.69, 4.14, and 3.99 ppm ((*H_b_* + *H_e_*), (*H_g_*), (*H_d_*), (*H_c_*), and (*H_f_*), respectively). These resonances chemically deviate to higher fields when the isosorbide unit is linked to the DD moiety (Figure S2 of supplementary material).

The knowledge of the real molar percentages of the 2,5-furandicarboxylate (%F) and 1,12-dodecanedioate (%DD) moieties effectively incorporated in the copolyesters’ backbone is of fundamental importance for the correct interpretation of structure-properties relationships. Therefore, the molar percentage of each diacid monomer effectively incorporated in the copolyester was assessed using ^1^H NMR relative integration areas (*I_a_* and *I_h_* as shown in Figure S2 of supplementary material), according to the following equations:

(1) $$\begin{array}{c} \%F=\left[\left(I_{a}/2\right)/\left(I_{a}/2+I_{h}/4\right)\right]\times 100,\\ \%\mathrm{DD}=\left[\left(I_{h}/4\right)/\left(I_{h}/4+I_{a}/2\right)\right]\times 100 \end{array}$$

Results summarized in Table 1 show that the real F/DD molar fractions are in acceptable agreement with the original molar feed ratios, despite some tendency to incorporate more DD units than the F ones. This is most probably due to the higher reactivity of DDA compared to DMFDC under the applied reaction conditions.

### 3.2. Crystallization Behavior and Thermal Properties

**Figure 2:**
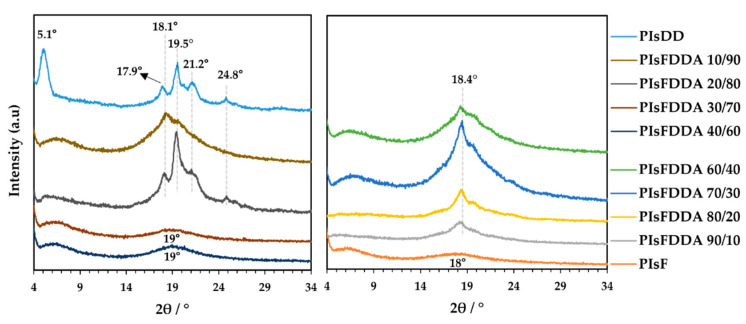
XRD patterns of PIsFDD copolyesters and related PIsF and PIsDD homopolyesters.

The XRD patterns of the novel PIsFDD 10/90 and 20/80 copolyesters, as well as the PIsDD homopolymer counterpart (Figure 2), i.e., those polymers incorporating higher amounts of the softer DD units, were revealed to have a semi-crystalline nature, showing accordingly five main diffraction peaks at 2*θ* = 5.1°, 17.9°, 19.5°, 21.2°, 24.8°, similarly to previously reported data [33]. Oppositely, in the case of the PIsFDD 60/40 and 40/60, the almost identical amount of two different repeating units, randomly incorporated into their backbone, resulting in an essentially amorphous character, with a halo centered at 2*θ* ≈ 19°. Nevertheless, a broad peak near 2*θ* ≈ 18.4° is detected for the 70/30 copolymer, evidencing some order. The very rigid PIsF and PIsFDD 90/10 (co)polymers are also essentially amorphous [34], most probably due to the inability of the very rigid chains to close pack and crystallize, whereas in the case of PIsFDD 80/20–70/30 copolymers, although a broad halo centered at the same angle (2*θ* ≈ 18°) is detected, they also show a broad peak evidencing some order.

**Figure 3:**
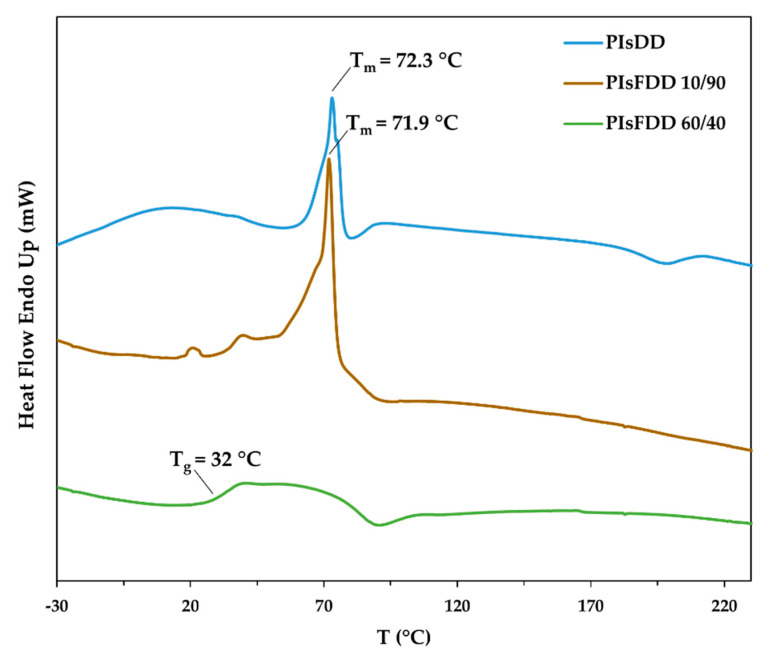
DSC heating traces of selected PIsFDD (co)polyesters.

In regard to the thermal behavior, the DSC traces displayed in Figure 3 and Figure S3 of Supplementary Material and relevant data summarized in Table 2 show, according to the semi-crystalline nature of PIsDD, a melting transition at 72.3 °C, whereas very similar or slightly lower values were obtained for the PIsFDD 10/90 and 20/80 copolyesters (71.9 and 70.5 °C, respectively).

Oppositely, the copolymers with higher amounts of stiff furan moieties (PIsFDD 70/30), showed no melting feature in their DSC traces, displaying only a glass transition slightly below 60 °C, in agreement with its amorphous character as highlighted before on XRD patterns.

PIsF is reported to be an amorphous polyester with a *T_g_* at approximately 157 °C [34], however, in the present study, by DSC analysis, this transition was not detected, but the DMTA trace showed a glass transition around 138 °C. Overall, as expected, the replacement of the very flexible DD fragment by the stiffer F structure induces an increase in *T_g_* by restricting the mobility of the chains. DMTA traces (Table 2 and Figure S4 of Supplementary material) clearly illustrate this, showing a drastic increase in *T_g_*, from −6.3 up to 137.9 °C, along with the F content increase and related stiffness. In line with previous results, we noticed that DMTA results tend to be higher than those obtained by DSC [35]. Moreover, in some of the tan δ traces, it was also possible to observe the β transition and the onset of the melting event.

**Figure 4:**
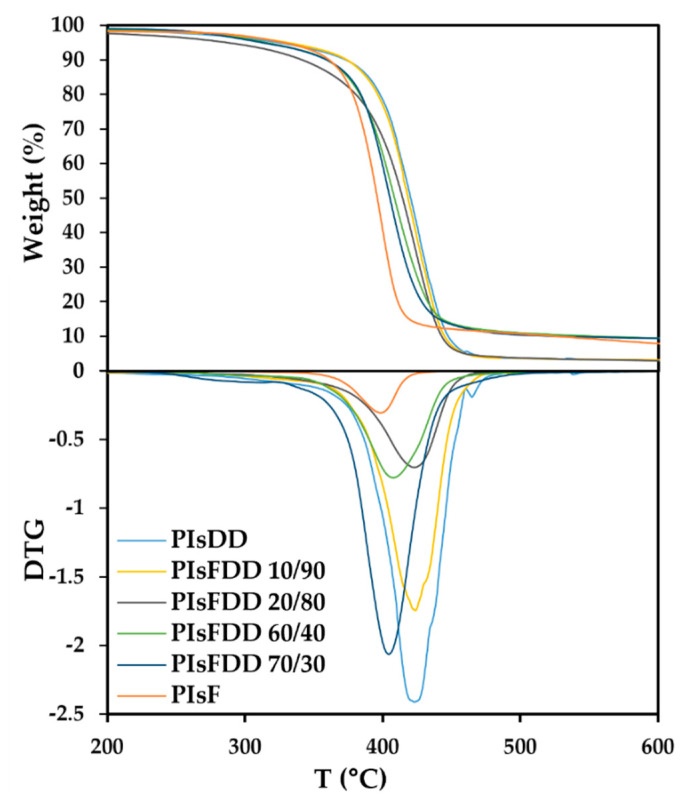
TGA and DTG thermograms of PIsFDDs and corresponding homopolyesters.

In order to assess the thermal stability of PIsFDD (co)polyesters, a TGA analysis of all polymers was carried out (Figure 4 and Table 2). The decomposition temperatures at 5% weight loss (*T_d,5%_*) and the maximum degradation temperature (*T_d,max_*) are summarized in Table 2. All copolyesters were thermally stable to at least up about 312 °C (*T_d,5%_*). They exhibited a single weight loss step with *T_d,max_* values ranging between 404 and 424 °C. T*_d,max_* of the PIsDD homopolymer is quite consistent with what has been reported before in the literature (*T_d,max_* = 423 °C) [25]. In general, PIsFDD (co)polyesters, with the exception of PIsFDD 20/80, showed higher thermal stability than PEF (*T_d,5%_* = 300 °C; *T_d,max_* = 398 °C) [32]. Furthermore, it was observed that with the increase in the amount of the DD moiety, there was an increase in *T_d,max_*_._ A similar trend was also reported before for similar polyesters [36].

### 3.3. Degradation Assays

**Figure 5:**
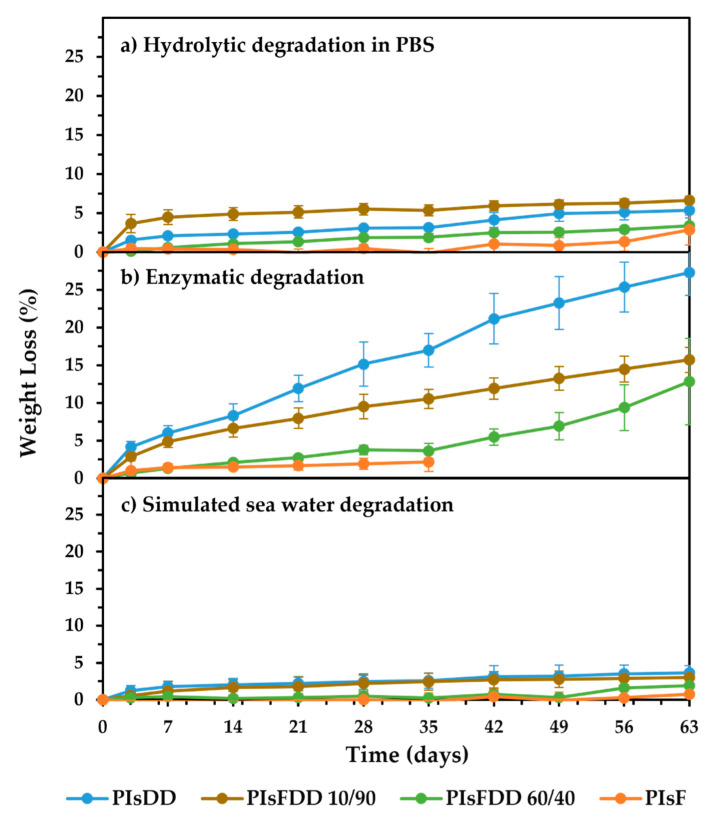
(Co)polyesters weight loss along time for hydrolytic degradation (**a**) in PBS; (**b**) under porcine pancreas; lipase-catalyzed degradation and (**c**) under simulated sea water conditions. Each value is the average of three weight measurements with standard deviation within 0.3−3.2%.

**Figure 6:**
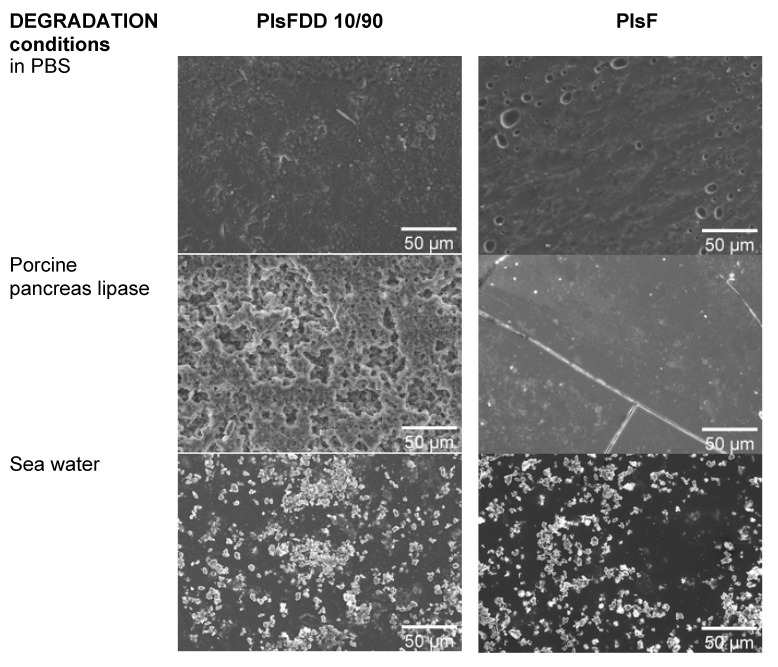
Comparison between typical SEM micrographs of PIsFDD 10/90 and PIsF after 63 days of degradation in PBS, enzymatic, and simulated sea water condition environments (1k times magnification).

To further investigate the potential of these biobased PIsFDD (co)polyesters and assess their (bio)degradation behavior under an aqueous environment, their degradability in three different conditions, namely: (a) in PBS at pH 7.4, (b) enzymatic degradation in the presence of porcine pancreatic lipase, at pH 7.4, and (c) simulated seawater conditions (sea salts solution at pH 8.2) were tested. These latter experimental conditions are quite relevant considering that today a high percentage of polymer debris ends up in aquatic environments, particularly in the ocean. Gravimetric (Figure 5) and SEM microscopy (Figure 6) analyses were used to study the (co)polyester films’ weight loss and morphology throughout the experiments, respectively.

The enzymatic degradation prompted the highest weight loss for all polymers studied, with values matching 27% weight loss after 63 days of incubation of PIsDD films, followed by PIsFDD 10/90 with ~16%. These results are in line with those previously reported for poly(1,20-eicosanediyl 2,5-furandicarboxylate) degradation studies under similar conditions [12] and enhanced compared to poly(lactic acid) (PLA) [11], which is quite promising.

Simulated seawater conditions were conducted to the overall lowest weight losses (around 3%), despite the slightly higher pH value. In this case, salinity must play a role. Therefore, one can anticipate the need for the correct disposal of these polymers after their use.

Overall, PIsF homopolymer showed the lowest weight loss (Figure 5); regardless of the conditions applied, at maximum, the weight loss was around 3% for pure hydrolytic conditions carried out in PBS for 63 days. Therefore, one can anticipate this high-*T_g_* polymer resistance to degradation under similar aqueous conditions. However, it is important to note that PIsF is a very brittle material that hampered enzymatic degradation gravimetric studies after 35 days of incubation due to the impossibility of handling the material.

Following a similar trend, it was found that, in general, increasing the F moiety content in the copolyesters backbone led to a decrease in weight loss in all degradation conditions, with the exception of PIsFDD 10/90 degradation under hydrolytic degradation in PBS, which loses more weight than PIsDD, despite the absolute value under enzymatic conditions is higher. This finding can be related to a lower crystallinity of PIsFDD 10/90 compared to PIsDD, which may facilitate water diffusion into the polymeric matrix, promoting increased hydrolytic ester cleavage. The same was not observed, however, with the enzymatic studies, probably because, in this case, the specificity of the enzymes also played a role. Lipases are well-known for their higher affinity for degrading polymers with aliphatic moieties rather than (hetero)aromatic ones [37].

The surface morphologies of (co)polyesters films before and after degradation in PBS, under porcine pancreas enzymatic degradation, and in simulated sea conditions were analyzed at the microscopic scale using SEM (Figure 6 and Figure S5 from supporting information).

Figure 6 clearly shows extensive changes in the surface morphology of the enzymatic treated PIsFDD 10/90 sample after 63 days of incubation compared to both pure hydrolytic conditions in PBS and in simulated seawater conditions, corroborating the weight loss findings. In particular, and also in accordance with the highest weight loss, PIsDD film also shows extensive degradation (Figure S5) under enzymatic conditions. Oppositely, in the case of simulated seawater conditions, degradation is almost imperceptible. Note that the cubic white spots detected on the film surface are due to sea salt crystals, obviously not observed for other conditions.

Based on these findings, the degradation under enzymatic conditions is obviously more severe than under hydrolytic conditions (PBS and simulated sea water), demonstrating that the porcine pancreas lipase has effectively contributed to the degradation of these polyesters.

## 4. Conclusions

In this work, aiming at obtaining fully biobased polyesters featuring a combination of good thermal properties with improved (bio)degradability, poly(isosorbide 2,5-furandicarboxylate-co-dodecanedioate) (co)polyesters series were successfully synthesized using the two-step bulk polymerization approach and typical reaction temperature for such systems (or even slightly lower than previously reported in the literature [17]). Their compositions, assessed by ^1^H NMR, were clearly controlled by the feed molar ratio. Depending on the F/DD moiety ratio, they acquired amorphous or semi-crystalline structures, and thermal properties varied accordingly. DMTA analysis showed that the glass transition values increased with the increasing amount of stiff furan moiety incorporated in the polymer backbone chain (from ≈ 0.2 °C to 73.8 °C) as expected for stiff furan ring-bearing polymers. Furthermore, all PIsFDDs had good thermal stability, as high as *T_d,5%_* ~ 330 °C, in line with PEF. Regarding their (bio)degradation, the enzymatic conditions lead to the highest weight loss compared to hydrolytic conditions (PBS and simulated seawater conditions), demonstrating that porcine pancreas lipase has effectively contributed to the degradation of these polyesters. Some degradation is, however, detected both under PBS incubation and simulated seawater conditions. Based on their properties, PIsFDDs stand out as promising biobased polymers with potential utility in environmentally friendly plastic applications. Further characterization, such as mechanical properties and degradation in soil, is planned for the future.

## Figures and Tables

**Table 1:** Feed (F/DD_feed_) and real (F/DD_real_) molar ratios of furan and 1,12–dodecanedioate moieties.

Polymer	F/DD_feed_ (mol%)	F/DD_real_ (mol%)	[*η*]/dL·g^−1^
**PIsDD**	0/100	0/100	0.20
**PIsFDD**			
**(10/90)**	10/90	6/94	0.18
**(20/80)**	20/80	12/88	0.17
**(60/40)**	60/40	47/63	0.17
**(70/30)**	70/30	62/36	0.12
**(80/20)**	80/20	76/24	0.13
**PIsF**	100/0	100/0	0.20

**Table 2:** Thermal properties of PIsFDD (co)polyesters with different compositions.

Sample	DSC		DMTA ^b^		TGA	
	*T_g_*/°C	*T_m_*/°C ^a^	*T_β_*/°C	*T_g_*/°C	*T_d,5%_*/°C	*T_d,max_*/°C
**PIsDD**	-	72.3	-	−6.3	323.9	423.0
**PIsFDD**						
**(10/90)**	9.0 ^a^	71.9	-	1.7	330.4	423.5
**(20/80)**	−8.5	70.5	-	0.2	286.7	422.7
**(60/40)**	38.8	-	−13.6	52.2	314.9	407.7
**(70/30)**	59.9	-	−11.3	73.8	312.1	404.0
**PIsF**	-	-	-	137.9	327.3	398.7

^a^ Assessed from the 1^st^ DSC heating scan, ^b^ Assessed from the maximum observed in the Tan δ vs. T curve, at 1 Hz.

## Data Availability

Not applicable.

## Supplementary Materials

The following supporting information can be downloaded at https://www.mdpi.com/article/10.3390/polym14183868/s1, Figure S1: ATR-FTIR spectra of all PIsFDD copolyesters studied and related PIsF and PIsDD homopolyesters, Figure S2: (A) Chemical structures of the triad units of the PIsFDD (co)polyesters. (B) ^1^H NMR spectra in CDCl_3_ of PIsFDD copolyesters and related PIsF and PIsDD homopolyesters, Figure S3: DSC heating traces of PIsFDD (co)polyesters, Figure S4: Tan δ traces of all studied polymers, at 1 and 10 Hz, Figure S5: SEM micrograph of PIsDD after 63 days of incubation with porcine pancreas enzyme (1.0 k magnification).
