# Supplementary material for: Isosorbide and 2,5-Furandicarboxylic Acid Based (Co)Polyesters: Synthesis, Characterization, and Environmental Degradation
Source: Polymers (Basel). 2022 Sep 15;14(18):3868. doi: 10.3390/polym14183868 (PMC9502350; doi:10.3390/polym14183868)
Supplement: Supplementary file 1 [file polymers-14-03868-s001.zip › polymers-1872623-supplementary.pdf]

polymers

Supplementary Materials

Isosorbide and 2,5-Furandicarboxylic Acid Based-(Co)Polyesters: Synthesis, Characterization, and Environmental Degradation

Chaima Bouyahya [1,2], Rafael Patrício [1], Ana Paço [3], Mafalda S. Lima [4], Ana C. Fonseca [4], Teresa Rocha-Santos [3], Mustapha Majdoub [2], Armando J. D. Silvestre [1] and Andreia F. Sousa [1,4,*]

[1] CICECO—Aveiro Institute of Materials, Department of Chemistry, University of Aveiro, 3810-193 Aveiro, Portugal
[2] Laboratoire des Interfaces et Matériaux Avancés, Université de Monastir, 5000 Monastir, Tunisia
[3] Centre for Environmental and Marine Studies (CESAM), Department of Chemistry, University of Aveiro, 3810-193 Aveiro, Portugal
[4] Centre for Mechanical Engineering, Materials and Processes, Department of Chemical Engineering, University of Coimbra Rua Sílvio Lima – Polo II, 3030-790 Coimbra, Portugal
* Correspondence: andreiafs@ua.pt

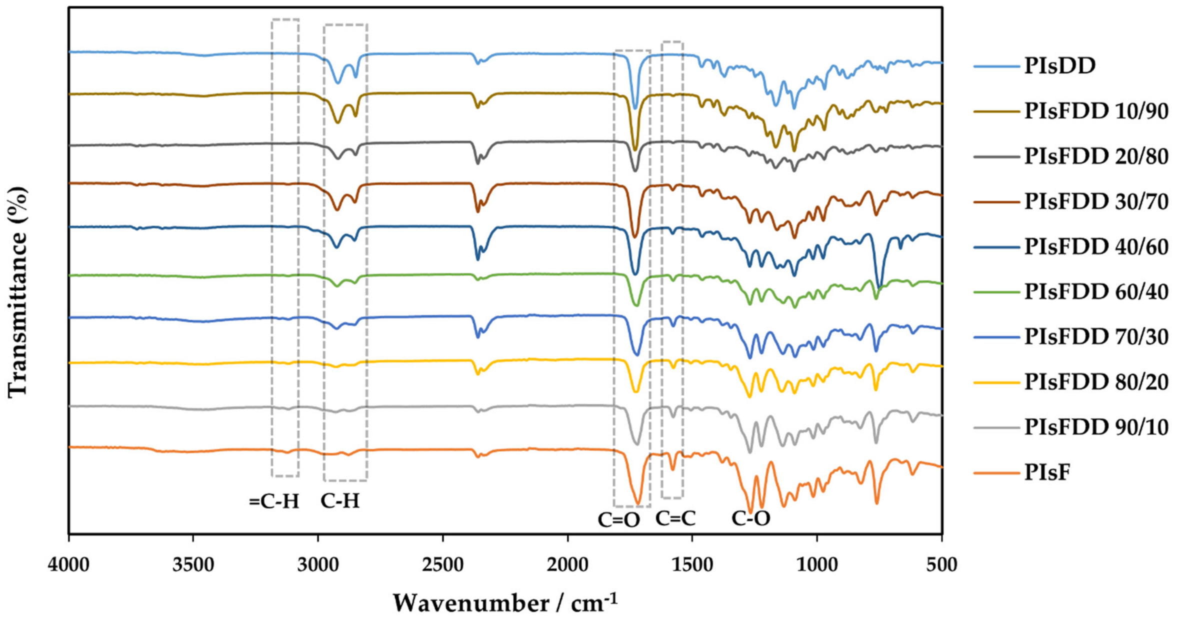

Figure S1. ATR-FTIR spectra of all PIsFDD copolyesters studied and related PIsF and PIsDD homopolyesters.

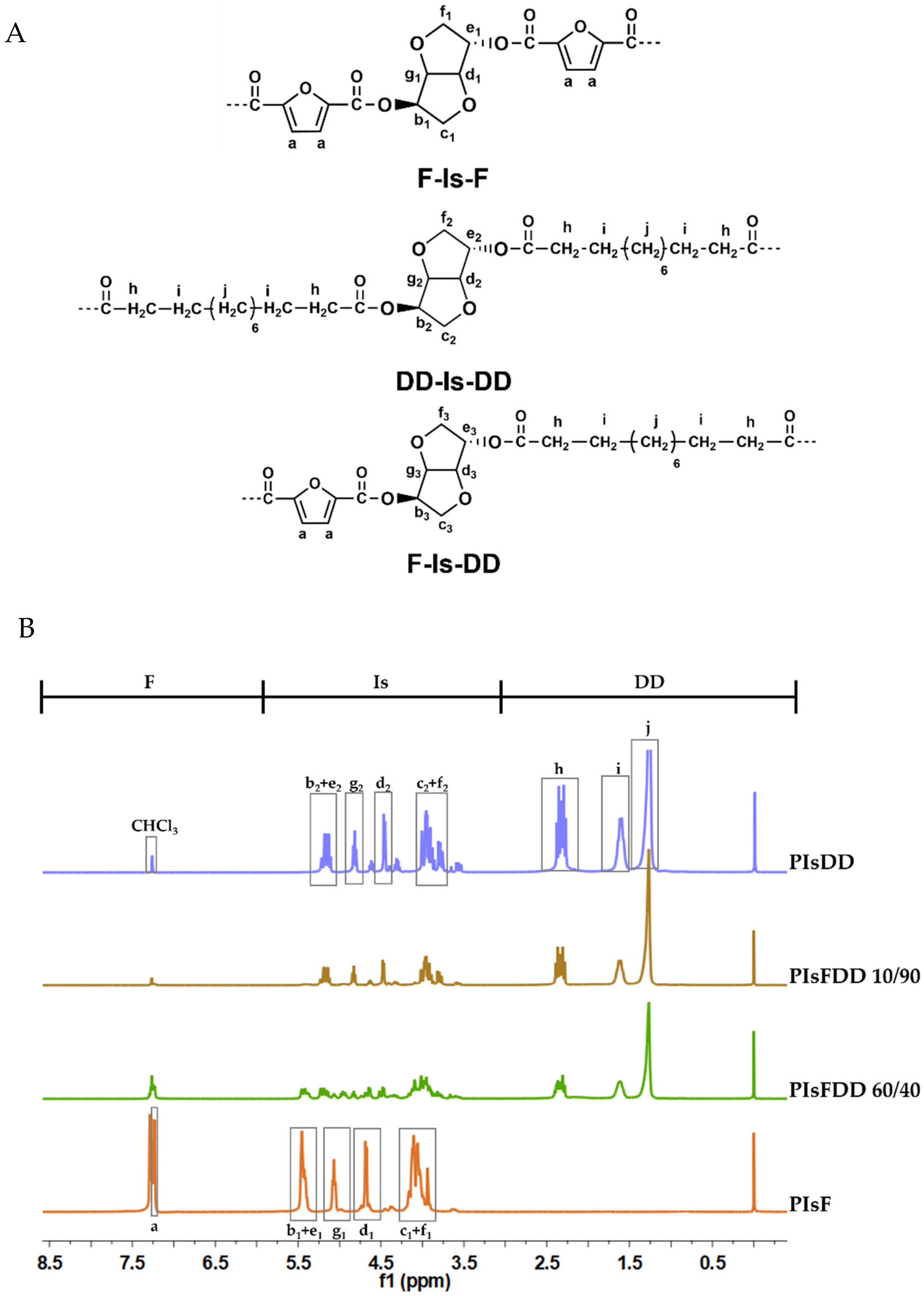

Figure S2. (**A**) Chemical structures of the triad units of the PIsFDD (co)polyesters. (**B**) ^{1}H NMR spectra in $CDCl_3$ of PIsFDD copolyesters and related PIsF and PIsDD homopolyesters.

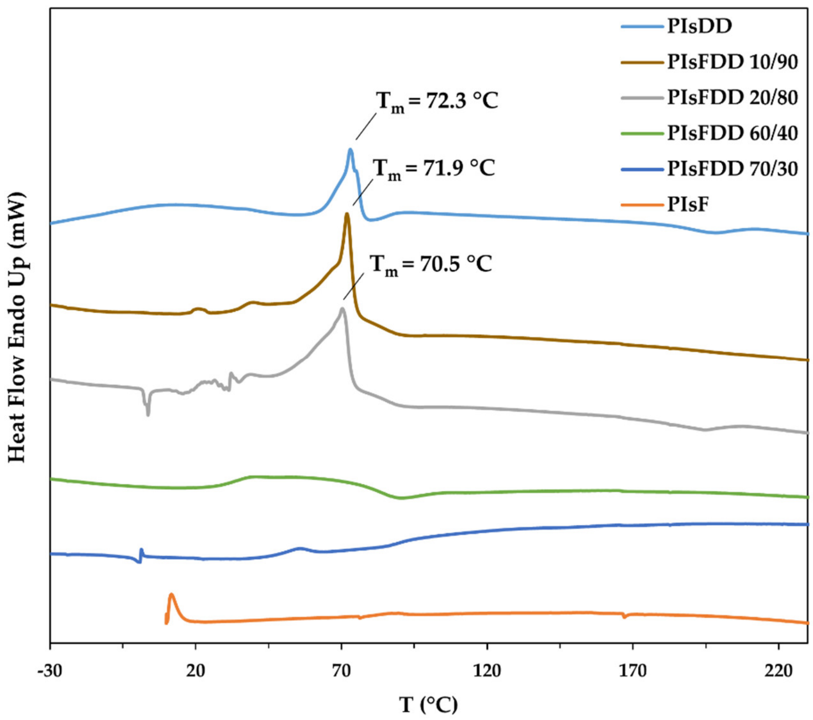

Figure S3. DSC heating traces of PIsFDD (co)polyesters.

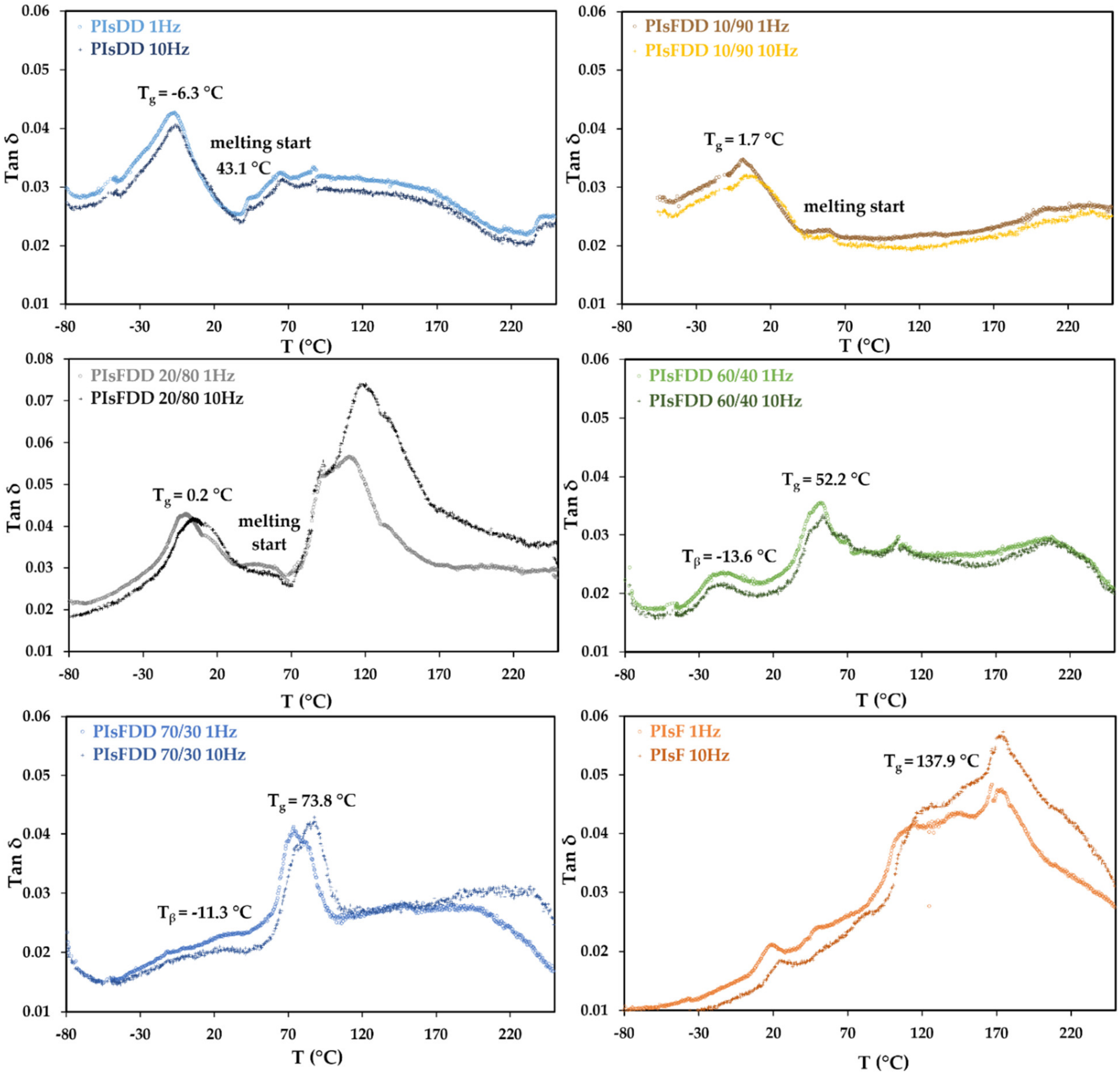

Figure S4. Tan δ traces of all studied polymers, at 1 and 10 Hz.

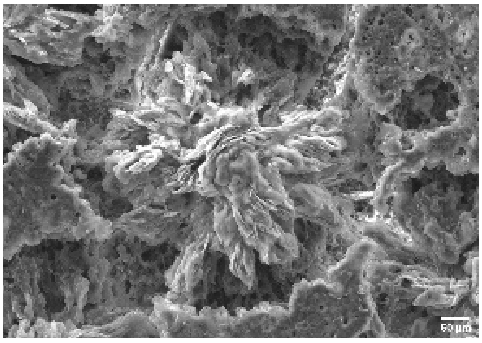

Figure S5. SEM micrograph of PIsDD after 63-days of incubation with porcine pancreas enzyme (1.0 k magnification).
